# Structure of the Central Nervous System

**Published:** 1891-03

**Authors:** 


*Structure of the Central Nervous System.* By Dr. LUDWIG EDINGER. Second Revised Edition. Philadelphia: F. A. DAVIS. 1890.

The object of these lectures was to lay before practitioners already familiar with the coarser anatomy of the brain what had been discovered in regard to its finer structure. Dr. Edinger has largely revised the first edition, incorporated the results of the intervening four years' work on the subject, and added several new figures.

The book opens with a short account of the methods of research adopted by different investigators, and of the embryology and comparative anatomy of the brain. The main facts of the general conformation of the brain, and of the convolutions as seen in dissections, are passed in review, with the histology of the different parts. Then follow descriptions of the course of the fibres in the cortex and white matter of the hemispheres, of the structure of the basal ganglia, the subthalamic region and base of the brain, with chapters on the roots of the peripheral nerves, the spinal cord and medulla and their constituent parts, and on the origins of the cranial nerves. The author has avoided the discussion of controversial matter as out of place in a work of the kind; and, with regard to disputed points, has adopted the views that, according to his own researches or the statements of the most trustworthy authors, seemed to be the correct ones.

The arrangement of the different sections is excellent, the descriptions clear and concise, so that the structure of the most complicated regions can be easily followed and understood. An abundance of diagrams and figures — 133 in number—form not the least important part of the book, and do what good illustrations should; namely, bring plainly before the reader's eye the points alluded to in the letterpress. From this point of view, the fact that some of the figures are a little roughly executed does not detract from their value.

Dr. Vittum, the translator, and Dr. Riggs, the editor, have done their work well, and they deserve thanks for putting in the hands of the English student a book which will give him a sound practical knowledge of the structure of the central nervous system, and be a useful guide in the dissection of the brain. If we were disposed to find fault, we should say that in a few instances the accepted English equivalent for the foreign nomenclature has not been given, and that expressions such as "back of" and "aside from" for "behind" and "apart from" do not, in our opinion, add either to the beauty or the richness of the English language.

